# Verification of *TREX1* as a promising indicator of judging the prognosis of osteosarcoma

**DOI:** 10.1186/s13018-016-0487-6

**Published:** 2016-11-24

**Authors:** Jinyi Feng, Ruilong Lan, Guanxiong Cai, Jinluan Lin, Xinwen Wang, Jianhua Lin, Deping Han

**Affiliations:** 1 Department of Central Laboratory, The First Affiliated Hospital of Fujian Medical University, Fuzhou, Fujian China; 2 Fujian Key Laboratory of Radiation Biology, Fujian Medical University, Fuzhou, Fujian China; 3 Fujian Key Laboratory of Individualized Active Immunotherapy, Fujian Medical University, Fuzhou, Fujian China; 4 Department of Orthopaedics, The First Affiliated Hospital of Fujian Medical University, Fuzhou, Fujian China

**Keywords:** *TREX1*, Osteosarcoma, Tumor stem cells, CD133, Prognosis evaluation

## Abstract

**Background:**

The study aimed to explore the correlation between the expression of *TREX1* and the metastasis and the survival time of patients with osteosarcoma as well as biological characteristics of osteosarcoma cells for the prognosis judgment of osteosarcoma.

**Method:**

The correlation between the expression of *TREX1* protein and the occurrence of pulmonary metastasis in 45 cases of osteosarcoma was analyzed. The CD133^+^ and CD133^−^ cell subsets of osteosarcoma stem cells were sorted by the flow cytometry. The tumorsphere culture, clone formation, growth curve, osteogenic and adipogenic differentiation, tumor-formation ability in nude mice, sensitivity of chemotherapeutic drugs, and other cytobiology behaviors were compared between the cell subsets in two groups; the expressions of stem cell-related genes *Nanog* and *Oct4* were compared; The expressions of *TREX1* protein and mRNA were compared between the cell subsets in two groups. The data was statistically analyzed. The measurement data between the two groups were compared using *t* test. The count data between the two groups were compared using *χ* ^2^ test and Kaplan–Meier survival analysis. A *P* value <0.05 indicated that the difference was statistically significant.

**Results:**

The expression of *TREX1* protein in patients with osteosarcoma in the metastasis group was significantly lower than that in the non-metastasis group. The difference was statistically significant (*P* < 0.05). Up to the last follow-up visit, the former average survival time was significantly lower than that of the latter, and the difference was statistically significant (*P* < 0.05). The expression of *TREX1* in human osteosarcoma CD133^+^ cell subsets was significantly lower than that in CD133^-^ cell subsets. Stemness-related genes *Nanog* and *Oct4* were highly expressed in human osteosarcoma CD133^+^ cell subsets with lower expression of *TREX1*; the biological characteristics identification experiment showed that human CD133^+^ cell subsets with low *TREX1* expression could form tumorspheres, the number of colony forming was more, the cell proliferation ability was strong, the osteogenic and adipogenic differentiation potential was big, the tumor-forming ability in nude mice was strong, and the sensibility of chemotherapeutics drugs on cisplatin was low.

**Conclusions:**

The expression of *TREX1* may be related to metastasis in patients with osteosarcoma. The expression of *TREX1* was closely related to the cytobiology characteristics of osteosarcoma stem cell. *TREX1* can play an important role in the occurrence and development processes. And, *TREX1* is expected to become an effective new index for the evaluation of the prognosis.

## Background

Pulmonary metastasis occurs in about 20% of the patients with osteosarcoma during the time of first visit. These are usually resistant to conventional chemotherapy drugs [1, 2]. A large number of clinical trials have proven that the biological characteristics of lung metastasis were not consistent with the primary tumor, thus leading to chemotherapy resistance [2–5]. Numerous evidence supported that osteosarcoma stem cells were the reason for pulmonary metastasis and chemotherapy drug resistance [6, 7]. Therefore, further study on the biological characteristics of osteosarcoma stem cells and its biochemical index are recommended to determine its relationship with pulmonary metastasis and chemotherapy drug resistance. This might be a precedent in the development of a specific targeted drug therapy; improving the prognosis and the overall survival of patients with osteosarcoma.

*TREX1* is main 3′–5′ exonuclease in mammalian animals. It has obvious preference for the special DNA sequence. The preference degree is related to the activity of exonuclease [8]. The present study has suggested that *TREX1* gene may be related to the immunity of tumor and lupus erythematosus [9–11] and the clinical outcome of cancer [12]. *TREX1* gene mutation or gene silencing can mediate DNA damage and cell death [13, 14]. Therefore, the low expression of *TREX1* gene may reduce the cytotoxicity of CTL and NK on tumor cells, so that the tumor cells occur to immune escape and the tumor growth can be promoted. However, it was unclear whether the expression of *TREX1* was related to the occurrence, development, and metastasis of osteosarcoma. It is necessary to clarify whether the osteosarcoma occurs to metastasis for the existing treatments and estimate the osteosarcoma condition using the specific indicator, in order to provide the diagnostic indicator for improving the clinical treatment.

The correlation between the expression of *TREX1*, clinical manifestation, and prognosis of osteosarcoma in patients as well as biological characteristics of osteosarcoma stem cell was investigated in the paper, the relationship between the expression of *TREX1* and osteosarcoma stem cells was clarified, so as to explore the correlation between the expression of *TREX1* and the metastasis risk and clinical prognosis of patients with osteosarcoma.

## Methods

### Patients, specimen, and data collection

Specimens from 45 cases of primary untreated osteosarcoma between January 2004 and March 2011 were included in this study. Inclusion criteria: after puncture or incision biopsy specimens, all patients of osteosarcoma were clearly pathological diagnosed by senior doctor of pathology. The standard preoperative neoadjuvant chemotherapy and postoperative chemotherapy for more than two courses of treatment were operated for all patients; all patients’ operation treatments were performed by the same senior doctors according to the patient Enncking staging. Chemotherapy with adriamycin + cisplatin + isofosfamide before operation, methotrexate + isofosfamide was adopted when the effect of the chemotherapy was poor. Postoperative chemotherapy was carried out according to the preoperative chemotherapy. Exclusion criteria: patients who did not conduct regular neoadjuvant chemotherapy or surgical treatment in our hospital for a variety of reasons.

In order to evaluate the development of local recurrence and distant metastases, following chemotherapy, all patients were undergoing lung checkup by bone scanning and lung CT scans every 3 months during the first 3 years after therapy and every 6 months thereafter.

The cohort included 23 males and 22 females, aged 8–54 years-old (mean, 21 years old). The formalin-fixed and paraffin-embedded surgical tumor samples were obtained from the archives of the Department of Pathology, First Affiliated Hospital, Fujian Medical University, Fuzhou, Fujian, China, for immunohistochemical staining. After reviewing medical records and contacting the patients or their relatives by telephone, follow-up information was available up to December 31, 2014. The use of tissue blocks and patient records was approved by the Ethics Committee of the First Affiliated Hospital, Fujian Medical University. The relevant clinical data included gender, age, tumor location, local recurrence status, distant metastasis status, and overall survival was recorded.

### Immunohistochemical method for the expression test of *TREX1* protein

Anti-*TREX1* monoclonal antibody (1:100, Abcam, USA) was used for the expression test of *TREX1* protein through immunohistochemical method. Known positive tissue specimens were used as positive control, single PBS was applied as the antibody for the negative control. Independent double-blind identification was applied for every slice by two pathologists, ten representative high-power field of vision was randomly selected and each field of vision counted about 200 cells. And then, the positive and negative expressions of *TREX1* protein were decided through the percentage of stained cells.

### Cell lines and culture

The human osteosarcoma cell lines MNNG/HOS, MG-63 and U-2 OS, which were purchased from the Chinese Academy of Sciences (Shanghai, China) cell bank and were used within 10 passages. They were placed in DMEM culture medium, included 10% fetal calf serum, 100U/mL penicillin and 100 μg/mL streptomycin, and placed in 75-mL flasks with filtered valves. All of the cells were incubated at 37 °C in 5% CO_2_, and the medium changed every other day. After confluence, cells were subdivided into new flasks for other experiments.

### Flow cytometry and cell sorting

Cells were detached using 0.02% Trypsinization-EDTA in phosphate-buffered saline (PBS), washed by PBS, and then incubated with fluorescent-labelled monoclonal antibodies human CD133/1 PE conjugated (Miltenyi, German) or respective isotype (mouse anti-human CD133 PE conjugated) controls. After washing steps, the labelled cells were sorted by flow cytometry (Becton and Dickinson, USA), collecting cells with CD133^+^ and CD133^−^ respective, for spheres, growth analysis, monoclonal formation, and the sensitivity of chemotherapy.

### Tumorspheres

Cells were seeded in a 6-well plates for a density of 1.0 × 10^6^cells/well and cultivated as spheres in DMEM-F12(Life Technologies medium with B-27 (2%; Invitrogen), L-gln(1×; Invitrogen), Life (20 ng/mL; Invitrogen), human EGF (20 ng/mL; Invitrogen) and human bFGF (20 ng/mL; Invitrogen). We use fresh medium every other day, and after culturing for 2~3 days, spheres were visible at inverted phase-contrast microscope (Olympus, Japan).

### Cell proliferation

Cell were plated in 96-well plates at a concentration of 3.0 × 10^3^ cells/well. After allowing cells to adhere, plates were collected at 1, 2, 3, 4, 5, 6, 7, and days. The number of living cells was quantificationally detected by MTT assay (3-(4,5-dimethylthiazol-2-yl)-2,5-diphenyltetrazolium bromide), which moved the medium and then added MTT 150 μL/well, after being incubated at 37 °C for 4 h and put DMSO 20 μL/well for 15 min later, was measured by micro-plate reader (Bio Med) at a wavelength of 490 nm with background subtraction at 570 nm. Experimental conditions were executed in triplicate and experiments repeated three times.

### Clonogenic assay

An equal number of cells were plated into 6-well plates at a concentration of 8.0 × 10^2^ cells/well. After 10 to 14 days of incubation, cells were fixed and stained with crystal violet, colonies consisting of at least 50 cells were enumerated for each group.

### Multilineage differentiation studies

Cells were plated at a density of 5.0 × 10^4^ cells/well in 24-well plates. For osteogenic differentiation, cells were incubated in the presence of 10 mM β-glycerol phosphate and 100 μg/mL ascorbic acid for the indicated times; the induction medium was changed every 3 to 4 days. Osteogenic differentiation was assessed after 21 days of incubation. Cells were fixed with 4% formaldehyde and stained with 2% Alizarin red S (Sigma) to visualize the formation of calcium deposits. To induce adipogenic differentiation, cells were incubated in the presence of 100 nM dexamethasone, 250 μM iso-butyl-methyl-xanthine (IBMX), 100 μM indomethacin and 10 μg/mL insulin. To visualize adipogenic differentiation, cultures were fixed in 4% formaldehyde and stained with Oil Red O (Sigma; 3 mg in 60% isopropanol) after 14 days in adipogenic induction medium. Images were taken using an inverted fluorescence microscope (Nikon, Eclipse TS 100).

### Xenograft model

Sorted cells were collected and resuspended at 1.0 × 10^4^ cells per 50 μL PBS, 50 μL of Matrigel (BD Biosciences) was added to each aliquot, and the cell-Matrigel suspensions were subcutaneously injected into the dorsum of 3- to 4-week-old non-obese diabetic/severe combined immunodeficiency (NOD/SCID) nude mice while under anesthesia.

### Quantitative real-time-PCR (qPCR) assay

Cellular RNA from different cell lines was collected using the RNeasy mini Kit (Tiangen). Primers and Taqman probe for detecting *TREX1* mRNA were designed by Beacon Designer (V.3.0, Premier BioSoft) and the sequences are in Table 1. Real-time PCR reaction products were added in appropriate primer pairs and then quantified by incorporated with SYBR Green I (Promega).

**Table 1:** Primer pairs used in qPCR

Gene	Forward primer 5′-3′	Reverse primer 5′-3′	Length
*TREX1*	5′-CCACTCCTTTCCTTACCACATC-3′	5′-CCACTCCGCCAAACAGAT-3′	106 bp
*Nanog*	5′-ACCTATGCCTGTGATTTGTGGG-3′	5′-AGAAGTGGGTTGTTTGCCTTTG-3′	169 bp
*Oct4*	5′-CTGAGGTGCCTGCCCTTCTA-3′	5′-CCAACCAGTTGCCCCAAAC-3′	166 bp
*Bglap*	5′-CTCACACTCCTCGCCCTATT-3′	5′-CGCCTGGGTCTCTTCACTAC-3′	143 bp
*adipoQ*	5′-CCCATTCGCTTTACCAAGAT-3′	5′-GGCTGACCTTCACATCCTTC-3′	139 bp
GADPH	5′-ATGACATCAAGAAGGTGGTG-3′	5′-CATACCAGGAAATGAGCTTG-3′	177 bp

### Western blot analysis

Total cellular proteins were harvested from incubated cells in lysis buffer. The concentrations of proteins were detected by BCA Protein Assay Reagent Kit (Sangon). Western blot analysis was performed by SDS-PAGE which was done in 10% glycine gels (Bio-rad) by loading 50 μg of total proteins per lane. After electrophoresis, fractioned proteins were transferred to a nitrocellulse membrane and blocked with 5% BSA (Bovine Serum Albumin) in TBST buffer for 1 h. And then, the memberane was incubated with a rabbit polyclonal primary antibody anti-*TREX1* and anti-β-actin (USA; dilution 1:1000) in blocking solution overnight at 4 °C. And then a goat anti-rabbit IgG monoclonal antibody (dilution 1:2000) conjugated with primary antibody at room temperature with gentle shaking for 1 h. Protein bands were added enhanced chemiluminescent (ECL) kit and detected immunoreactivity by exposure to autorads.

### Statistical analyses

All of the data was statistically analyzed through SPSS 17.0 software. The measurement data between the two groups were compared using *t* test. The count data between the two groups were compared using *χ* ^2^ test and Kaplan–Meier survival analysis. *P* < 0.05 indicated that the difference was statistically significant.

## Results

### Three-year follow-up results

The patients were divided into the metastasis group (*n* = 25) and non-metastasis group (*n* = 20) according to the distant metastasis to lungs, spine and retroperitoneum within 3 years. Among them, seven patients were observed with metastasis at time of preliminary diagnosis, accounting for 15.6% (7/45) of the total patients. The metastasis time spanning in the metastasis group was from preliminary diagnosis to postoperative 20 months, averagely about 6.8 months. Average death time was 24.4 months for the metastasis group, but three patients exceeded 36 months accounting for 12% (3/25). Only one patient had the longest survival time of 67 months, accounting for 4% (1/25). On the other hand, the average survival time in the non-metastasis group was 52.6 months at the end of the 3-year follow-up visit.

### HE staining of osteosarcoma tissue in two groups

**Fig. 1:**
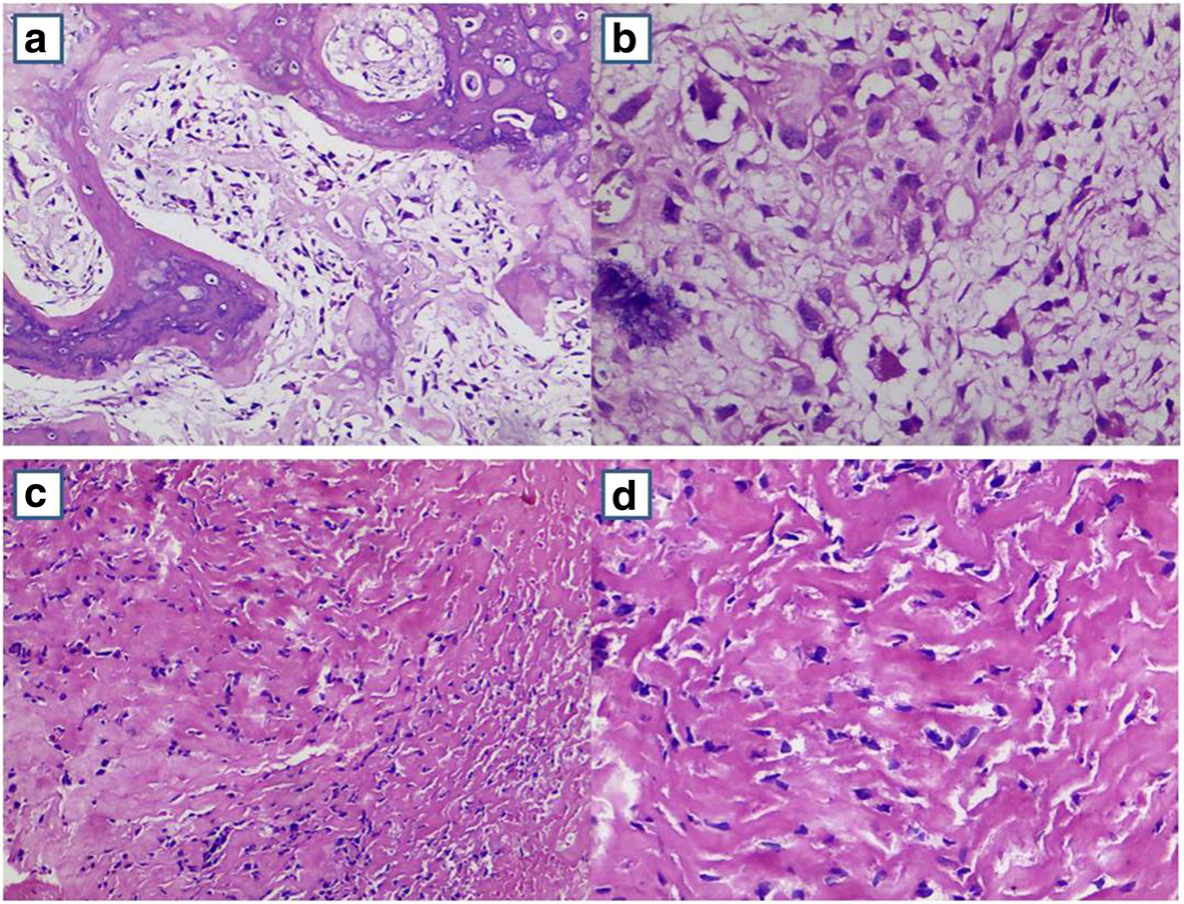
The HE staining of osteosarcoma tissue of the non-metastasis group (**a**, **b**) and metastasis group (**c**, **d**) with 100× (**a**, **c**) or 200× (**b**, **d**)

In the non-metastasis group, the low power lens showed the naive osteosarcoma tumor cells were diffusely distributed and the marrow cavity was infiltrated. Moreover, the lace-like immature osteoid tissue was observed. The high-power lens showed that the tumor cells were pleomorphic, polygonal, round, and irregular with the clear boundary. The cytoplasm was abundant, slightly basophilic and transparent. The cell nuclei were unequal-sized. The dicaryon was observed in some cells. The shape was irregular. The chromatin was coarse granular. 1~4 small nucleolus were observed. The nuclear fission was observed. The mesenchyma was mucoid. A small amount of osteoid tissue was scattered in the mesenchyma (Fig. 1a, b). The heterogenic tumor cells were observed in the osteosarcoma tissue in the metastasis group under low power lens. A large number of immature osteoid tissues were observed in the surrounding. They were parallel and irregular. No lining osteoprogenitor or small vessel was observed. Small amount of atypical cells were scattered surrounding the immature osteoid tissue under high power. The cells were irregular in shape. The nucleoplasm ratio was relatively large. The chromatin was fine and smooth. No clear nucleolus was observed. The mitotic phenomenon was rare (Fig. 1c, d).

### Expression of *TREX1* protein in osteosarcoma tissues in two groups

**Fig. 2:**
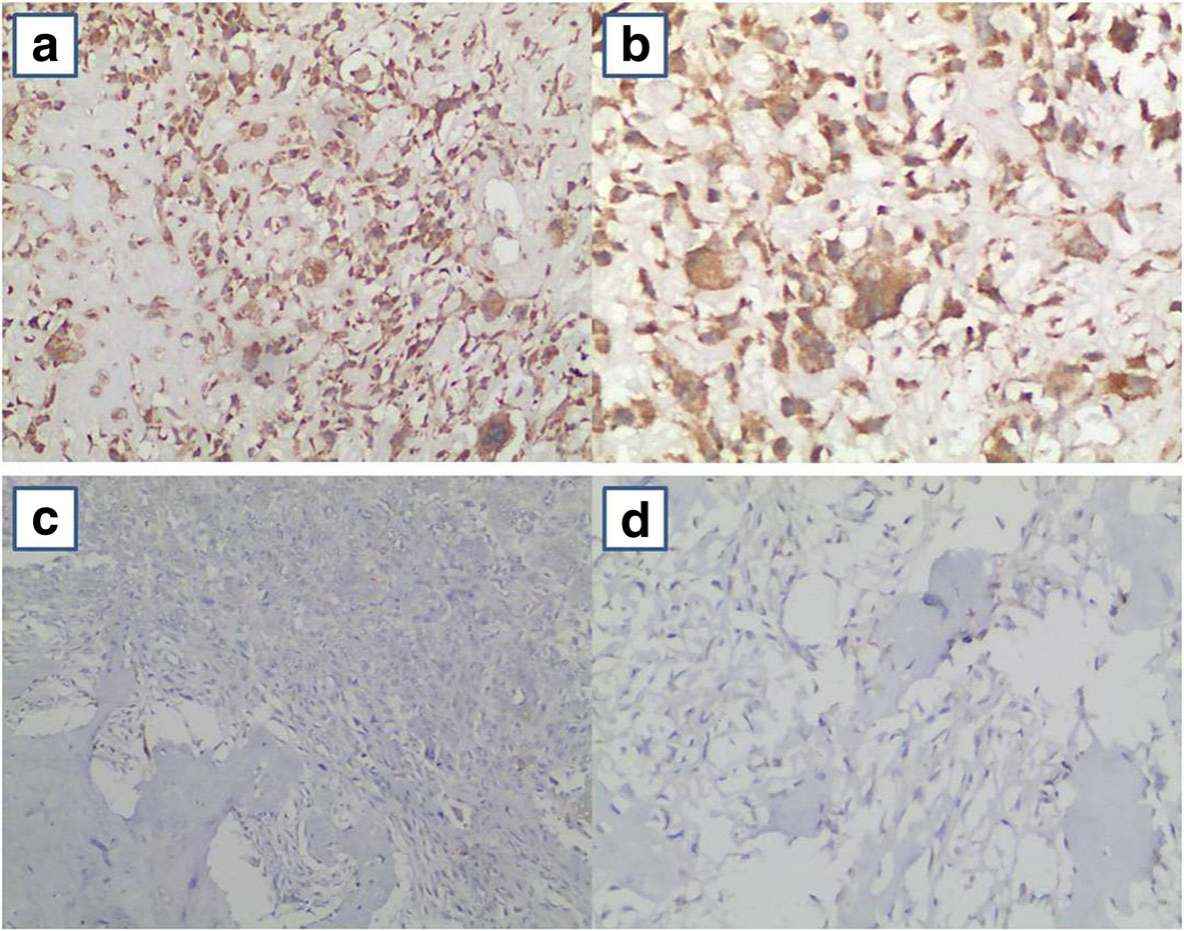
The expressions of TREX1 protein of osteosarcoma tissue of the non-metastasis group (**a**, **b**) and metastasis group (**c**, **d**) with 100× (**a**, **c**) or 200× (**b**, **d**)

*TREX1* protein was expressed in the cytoplasm and nucleus in the osteosarcoma tissues of the non-metastasis group. It was brown yellow to dark yellow granular, diffusely and scatteredly distributed (Fig. 2a, b). The *TREX1* protein observed in cytoplasm was faint yellow granular in the osteosarcoma tissue of the metastasis group, scatteredly distributed. *TREX1* protein was not expressed in the nucleus (Fig. 2c, d). The analysis by IPP6.0 software showed that the positive rate of the non-metastasis group was high, the positive intensity was strong and the difference was statistically significant (*P* < 0.05), suggesting that *TREX1* protein was highly expressed in the osteosarcoma in the non-metastasis group.

### Relationship between *TREX1* expression and clinicopathological factors in patients with osteosarcoma

There are many clinicopathologic characteristics for the tumor tissue. All of 45 samples were divided into the high expression group (*n* = 21) and low expression group (*n* = 24) according to the expression of *TREX1*; the *P* value was statistically analyzed, in order to determine the correlation between the patients’ age, gender, site, histological type, pathological stage, distant metastasis, other clinicopathological factors and the expression of *TREX1*. Table 2 shows that the expression of *TREX1* is not significantly correlated with the patients’ age, gender, site, histological type or other clinicopathological factors (*P* > 0.05); the higher the pathological stage possessed the lower the expression of *TREX1*, suggesting that the *TREX1* was significantly correlated with the Enneking stage of osteosarcoma and the expression of *TREX1* protein in the metastasis group was significantly lower than that of non-metastasis group (*P* < 0.05).

**Table 2:** Expression of *TREX1* protein in human osteosarcoma tissue and its relation with the clinical biological behavior of osteosarcoma

Clinical data	High expression (*n* = 21)	Low expression (*n* = 24)	*P*
Gender			0.554
Male	12	11	
Female	9	13	
Age			0.729
<30	17	18	
≥30	4	6	
Position			0.441
Femoral	8	12	
Tibia	10	7	
Others	3	5	
Enneking staging			0.000
I	6	–	
IIA	14	10	
IIB	1	10	
III	-	4	
Histologic type			0.785
Osteoblastic	10	9	
Chondroblastic	7	8	
Fibroblastic	3	4	
Others^a^	1	3	
Distant metastasis			0.000
Yes	4	21	
No	17	3	

^a^Others (telangiectatic, small cell, parosteal, undifferentiated osteosarcoma)

### Kaplan–Meier survival analysis of *TREX1* and prognosis of patients with osteosarcoma

**Fig. 3:**
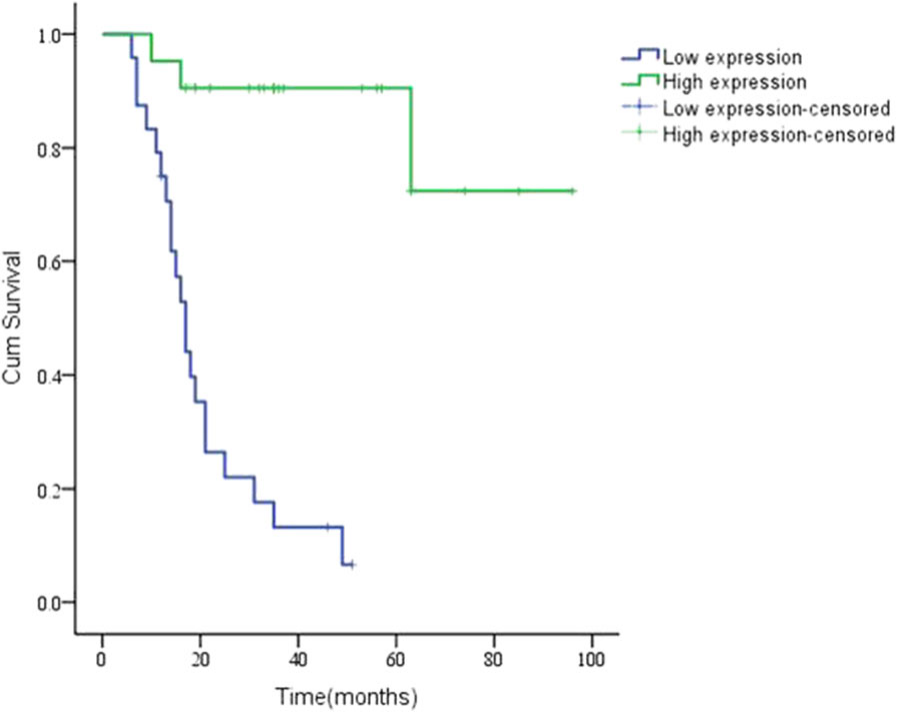
Kaplan–Meier curves displaying the survival of patients with different expression levels of *TREX1* protein

The single factor analysis of 45 patients with osteosarcoma showed that the expression of *TREX1* was an independent factor affecting the patients’ prognosis (*P* < 0.05). The expression of *TREX1* was positively related to the prognosis of patients with osteosarcoma, namely the higher the expression of *TREX1* was, the longer the patients’ survival time (Fig. 3).

### Sorting of human osteosarcoma stem cells

**Fig. 4:**
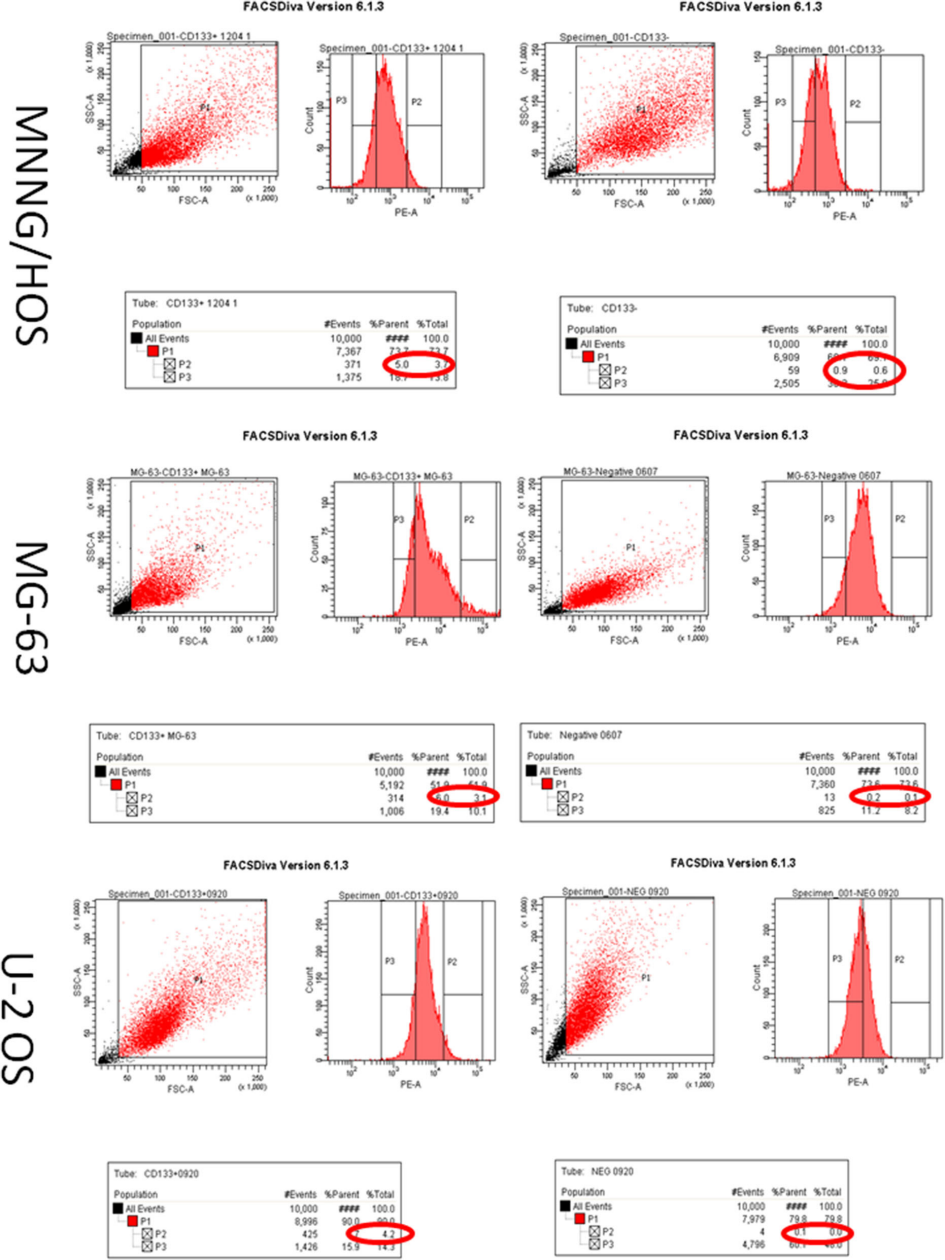
Sorting of human osteosarcoma cells. *Left* the cell subsets with the detection of CD133^+^ (P2). *Right* were the isotype controls

The proportions of fluorescent P2 cells subset (CD133^+^ cell subset) in MNNG/HOS, MG-63 and U-2 OS cell lines fluctuated in 1~5%, averagely about 3%. The difference was not statistically significant between the cell lines (*P* > 0.05), as shown in (Fig. 4). This suggested that the proportions of CD133^+^ cell subsets were roughly equal in human osteosarcoma cell lines, namely that the proportion of osteosarcoma stem cells was constant. Any cell line can be selected to study the biological characteristics of osteosarcoma stem cell as a research carrier. Considering that the stem cells sorted by osteosarcoma MNNG/HOS cell formed the tumor earlier and the tumor was larger, thus, osteosarcoma cells line MNNG/HOS CD133^+^ cell subset was selected as the research object.

### Tumorsphere culture after isolation of human osteosarcoma stem cells

**Fig. 5:**
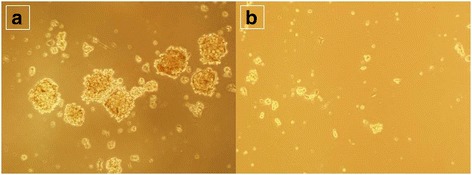
The contrast of the formation abilities of tumorsphere of CD133^+^ cell subsets (**a**) and CD133^−^ cell subsets (**b**) (100×)

The tumorsphere was cultured in the serum-free stem cell medium. CD133^+^ cells aggregated into clusters after incubation for 24 h. The tumorspheres were gradually increased. The tumorsphere was not increased significantly 7 days later (Fig. 5a). While, CD133^−^ cells were in single state in after 24 h incubation. There was no significant tumorsphere after culture for 7 days (Fig. 5b). Only scattered cells aggregating into small clusters were found, which was just equivalent to the tumor volume of the culture of CD133^+^ cells subset for 24 h. The difference was statistically significant (*P* < 0.05). The results showed that the CD133^+^ cell subset had obvious stem cell characteristics, had higher rate and larger volume of tumor-forming and the proliferation was rapid.

### Biological characteristics of human osteosarcoma MNNG/HOS stem cell subsets

**Fig. 6:**
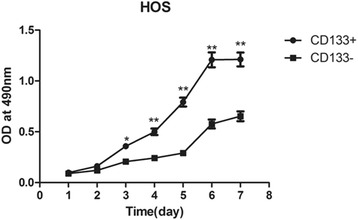
The proliferation of CD133^+^ and CD133^−^ cell subsets in human osteosarcoma MNNG/HOS cells (MTT assay)

MTT assay showed that CD133^+^ cell subsets entered logarithmic phase after inoculation for 3~7 days. The OD values of CD133^+^ cell subsets were higher than that of CD133^−^ cell subset and the difference was statistically significant (*P* < 0.05), as shown in (Fig. 6), suggesting that the growth and proliferation abilities of CD133^+^ cell subsets in osteosarcoma MNNG/HOS cells were stronger.

**Fig. 7:**
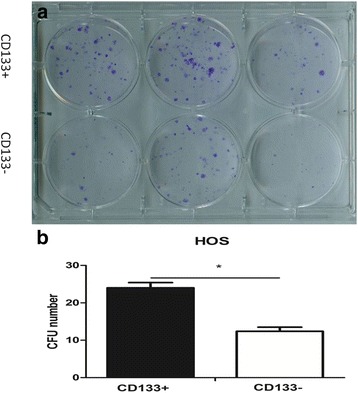
The contrast of the clone forming ability (**a**: sorted cells; **b**: the statistical analysis) of CD133^+^ and CD133^−^ cell subsets in human osteosarcoma MNNG/HOS cells

CD133^+^ cell subset sorted from human osteosarcoma MNNG/HOS cells had a strong ability of clone formation. The sorted cells were inoculated, respectively, in two groups, which were repeated for three times (Fig. 7a). The number of colonies was calculated after culture for 2 weeks. The mean number of total clone was counted. The clone number of CD133^+^ cell subsets was (24 ± 3.16), which was significantly higher than that of the CD133^−^ cell subset (12 + 2.41). And, the difference was statistically significant between the two groups (*P* < 0.05) (Fig. 7b).

**Fig. 8:**
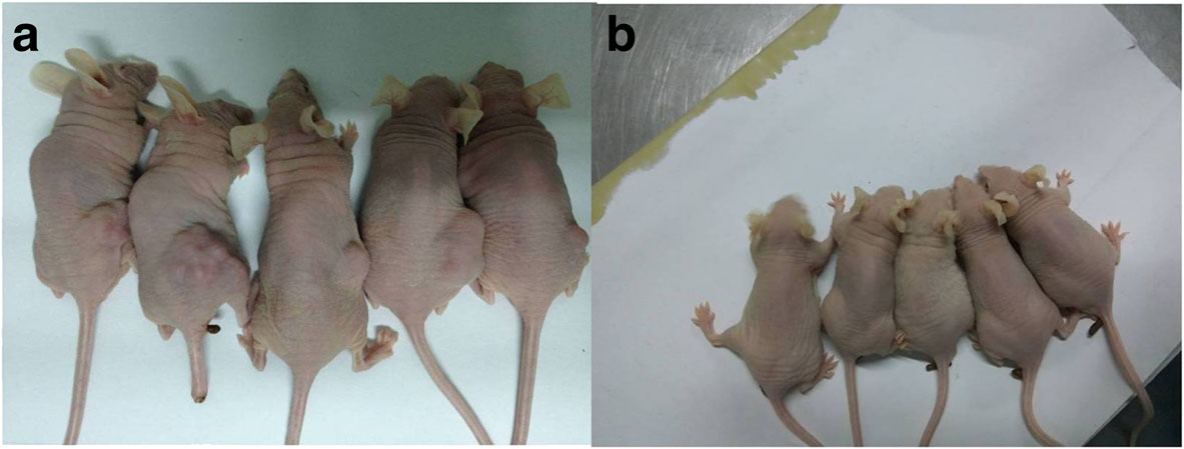
The tumor growth of the inoculation of human osteosarcoma CD133^+^ (**a**) and CD133^−^ (**b**) cell subsets in nude mice

After CD133^+^ cell subsets in human osteosarcoma MNNG/HOS cells were inoculated subcutaneously in nude mice, the tumor easily formed. After 1.0 × 10^4^ cells were inoculated for 12 days, small nodules formed. And, the tumor increased and formed obviously in 4 weeks (Fig. 8a). While no obvious tumor growth was observed in CD133^−^ cell subset inoculation area in the control group with the same cells inoculated in 4 weeks (Fig. 8b).

### Osteogenic and adipogenic induction

**Fig. 9:**
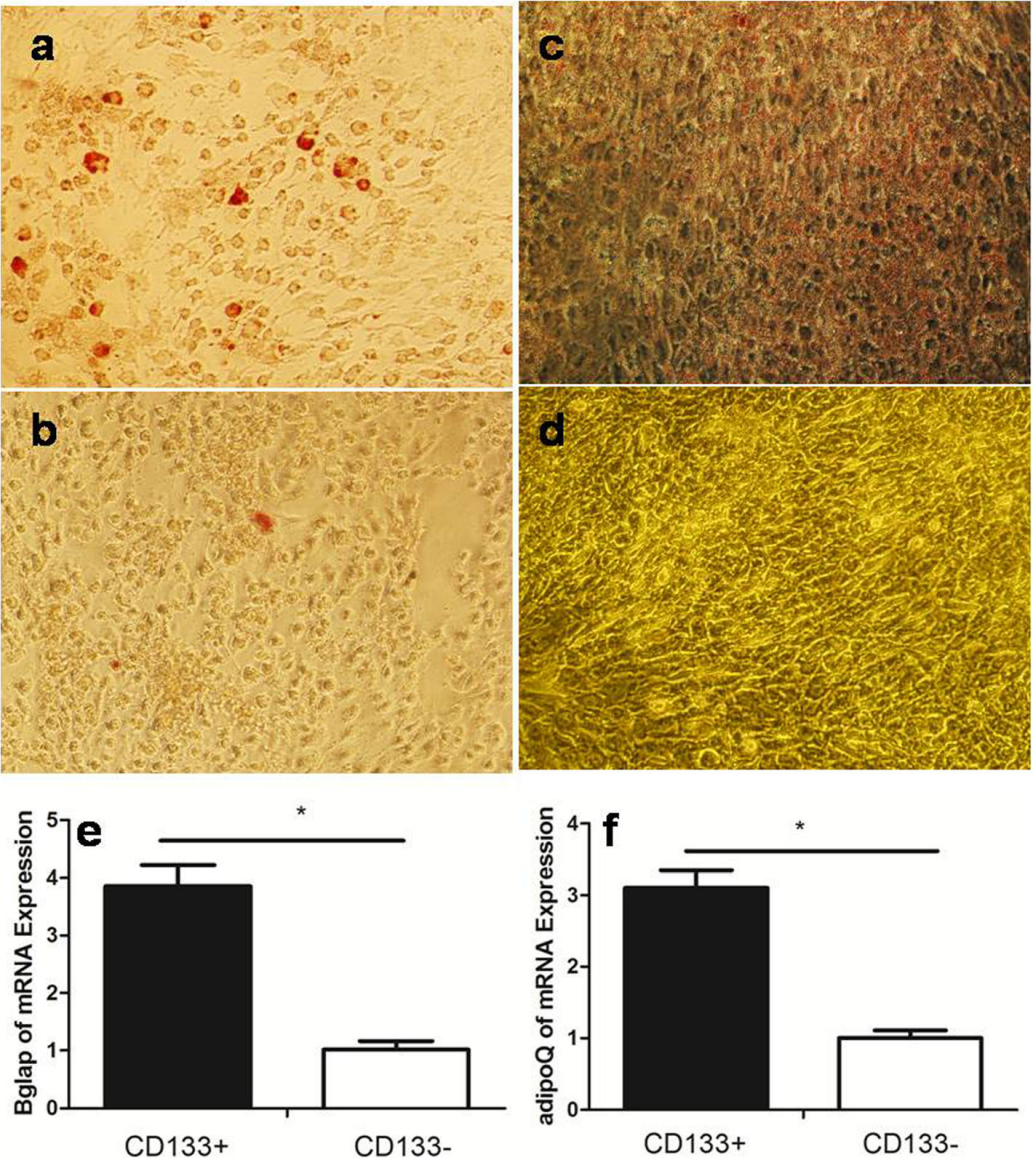
The osteogenic induction (**a**, **b**), adipogenic induction (**c**, **d**), and expressions of osteogenesis gene *Bglap* (**e**) and adipogenic gene *adipoQ* (**f**) of CD133^+^ (**a**, **c**) and CD133^−^ (**b**, **d**) cell subsets in human osteosarcoma MNNG/HOS cells

After the osteogenic induction of CD133^+^ cell subsets in human osteosarcoma MNNG/HOS cells for 18 days, a lot of calcium nodules were investigated through Alcian staining (Fig. 9a), while only individual calcium nodules can be found in CD133^-^ cell subsets (Fig. 9b). Similarly, after the adipogenic induction of the former for 21 days, a large number of small lipid droplets were found through oil red staining (Fig. 9c), while the lipid droplet was not found in the latter (Fig. 9d). The expressions of osteogenesis gene *Bglap* and adipogenic gene *adipoQ* in CD133^+^ and CD133^−^ cell subsets were detected, respectively, by qPCR. The results showed that the mRNA expressions of *Bglap* and *adipoQ* were more significantly increased in CD133^+^ cell subset, about 3~4 times than that in CD133^-^ cell subset, and the difference was statistically significant (*P* < 0.05) (Fig. 9e, f), suggesting that the CD133^+^ cell subset had the significant differentiation potential of the characteristics of osteosarcoma of osteogenic and adipogenic.

### Chemotherapy sensitivity test

**Fig. 10:**
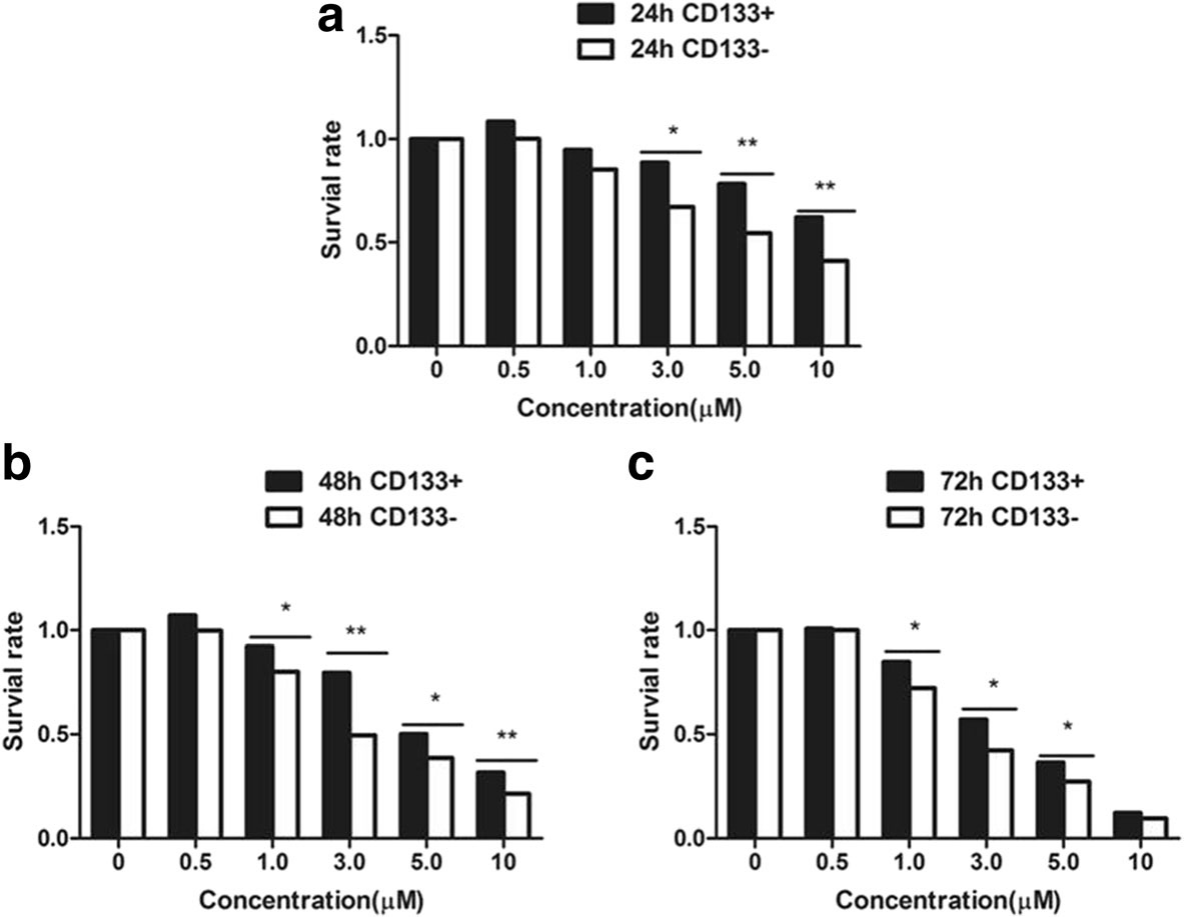
Effect of different concentrations of cisplatin on the survival rate of CD133^+^ and CD133^−^ cell subsets in human osteosarcoma MNNG/HOS cells in different time (**a**:24 h; **b**:48 h and **c**:72 h)

The human osteosarcoma MNNG/HOS cells were inoculated in 96-well plates and cultured. The OD values were measured at different time-points after cisplatin was added according to the concentration gradient. The OD values divided by the OD values in the negative control group were used as the survival rate (SR). The low concentration (0.5 μmol/L) of cisplatinum had almost no inhibiting effect on osteosarcoma cells in two groups. With the increase of cisplatinum concentration and the extension of drug action, the survival ratios in two subsets were decreased gradually, but the decrease of CD133^−^ cell subset was more significant. The difference was statistically significant (*P* < 0.05) when the concentration was ≤5 μmol/L for 72 h (Fig. 10a, b). When 10 μmol/L cisplatinum was used for 48 h, the difference was statistically significant (*P* < 0.05), while the difference was not statistically significant (*P* > 0.05) when the concentration of cisplatinum was 10 μmol for continuous 72 h (Fig. 10c).

### Expressions of osteosarcoma stem cells related genes *Nanog* and *Oct4*

**Fig. 11:**
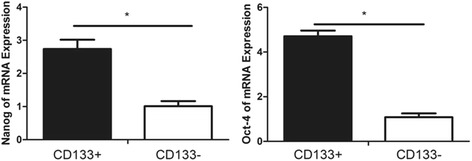
The expressions of stem cell-related genes *Nanog* and *Oct4* in CD133^+^ and CD133^−^ cell subsets of human osteosarcoma MNNG/HOS cell

The expressions of stem cell-related genes *Nanog* and *Oct4* in osteosarcoma MNNG/HOS were detected by qPCR. The expressions of *Nanog* and *Oct4* of CD133^+^ cell subset were higher than those of CD133^−^ cell subset, and the difference was statistically significant (*P* < 0.05), as shown in (Fig. 11).

### Expression of *TREX1* in human osteosarcoma MNNG/HOS cell subsets

**Fig. 12:**
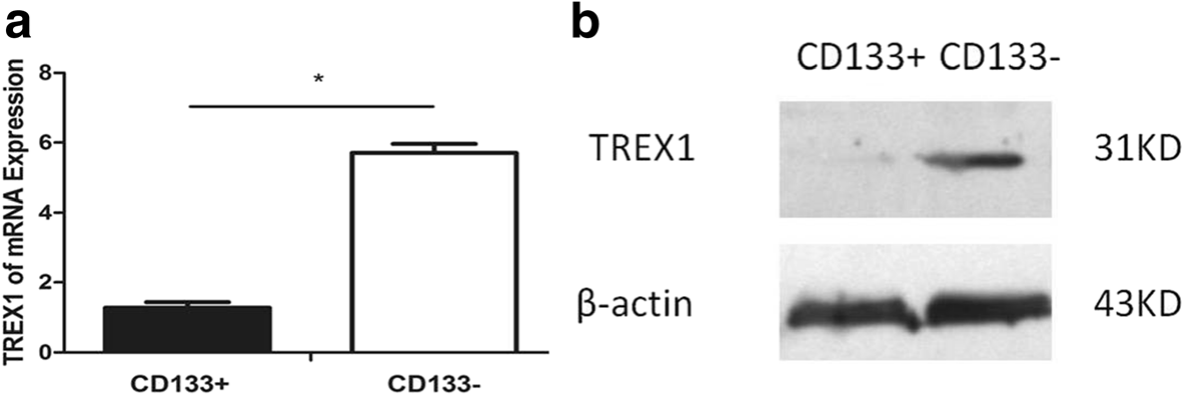
The expressions of *TREX1* mRNA (**a**) and protein (**b**) in CD133^+^ and CD133^−^ cell subsets of human osteosarcoma MNNG/HOS cell

The expression of *TREX1* mRNA in the sorted CD133^−^ cell subsets was 5~6 times of CD133^+^ cell subset by qPCR, and the difference was statistically significant (*P* < 0.05), as shown in (Fig. 12a). The protein was isolated and tested by Western blot. The grey level was calculated using the ImageJ software. The CD133^−^/CD133^+^ ratio was 2.5, namely the expression of *TREX1* protein in the sorted CD133^−^ cell subsets was 2.5 times of CD133^+^ cell subsets, and the difference was statistically significant (*P* < 0.05), as shown in (Fig. 12b), suggesting that the expression of *TREX1* in CD133^+^ cell subset with the nature of osteosarcoma stem cells was significantly lower than that of CD133^−^ cell subsets in non-osteosarcoma stem cells.

## Discussion

Osteosarcoma is the most common primary malignant bone tumor in children and adolescents [1]. Although neoadjuvant chemotherapy combined with surgical resection can improve the 5-year survival rate, the overall prognosis is unsatisfactory. Distant metastasis is the main cause of death in patients with osteosarcoma. Previous studies report that 20% of patients have distant metastatic lesions at the initial diagnosis. In contrast, 80% of patients develop metastasis after the surgery [15]. In this group, 15.6% (7/45) of the patients occurred metastasis in the preliminary diagnosis, which was similar with that of the literature [15]. More than half of the patients occurred to metastasis within 3 years after the operation (25/45). The follow-up visit found that the survival time in the metastasis group was significantly lower than that of non-metastasis group. The average time to metastasis was 6.8 months and with the average survival time of 24.4 months in the metastatic group. While the average survival time for the non-metastatic group was 52.6 months. The average survival time of the metastasis group with the discovery of metastasis was only 18 months. The result of follow-up visit showed that the metastasis time was different from the preliminary diagnosis to the postoperative 20 months in the metastasis group. Combined with the results that the metastasis time was about 18 months from the discovery of metastasis to death, the survival time of most patients was less than 3 years, and the number of patients whose survival time was more than 3 years in the metastasis group was only 12% (3/25) up to the end of the follow-up visit, the time boundary was established as 3 years in this work to facilitate the control study.

To improve the 5-year survival rate of patients with osteosarcoma, the metastasis of osteosarcoma must be effectively prevented. A large number of clinical trial proved that the metastatic tumors had multi-drug resistance to chemotherapy drugs. Lung metastatic tumor was inconsistent with the biological characteristics of primary tumor [2–5], resulting that the chemotherapy drugs being sensitive to the pathological type of primary osteosarcoma was insensitive even resistant to the metastatic tumor. Recent studies suggest that tumor stem cells may be the cause of drug resistance and metastasis of malignant tumors. Further study on the biological characteristics of tumor stem cells may help us to clarify the drug resistance mechanism of tumor, to develop the specific targeted drug and to improve the survival of patients with malignant tumor.

*TREX1* gene correlation with tumors has attracted the attention of scholars in recent years [16–19]. Dong et al. [20] analyzed the genotype and survival rate of over 700 patients with pancreatic cancer. The result found that *TREX1* gene could be applied as one of the important predictive factors for the overall survival rate of patients with pancreatic cancer. However, no study on TREX1 gene was reported on other solid tumors [18]. It is unclear if it is suitable as a predictive factor for other tumors. Immunohistochemistry experiment showed that the morphologies of tumor cells were irregular in 25 cases of patients with osteosarcoma. The nucleoplasm ratio was relatively large. The chromatin was fine and smooth. The nucleoli were not obvious. The nuclear fission was rare. *TREX1* protein was lowly expressed in 21 cases of patients in the metastasis group, suggesting that the expression of *TREX1* was closely related to the lung metastasis in osteosarcoma. Further analysis showed that the expression of *TREX1* was high in patients at stage I and most patients at stage II, while the expression of *TREX1* was low in patients at stage IIb and stage III, the expression of *TREX1* was related to the patients’ Enneking stage (*P* < 0.05). There was no significant correlation between the expression of *TREX1* and the type of osteosarcoma. The higher the expression of *TREX1* was the longer the survival time. Therefore, the expression of *TREX1* can be used as an effective index to jugde the prognosis of patients with osteosarcoma. However, the number of cases in this group is relatively few. More cases should be analyzed and followed-up longer in order to confirm the significance of this prognostic indicator. The cytology experiment in vitro on the correlation between the expression of *TREX1* and the biological characteristics of osteosarcoma stem cells was further studied.

The tumor stem cells can be isolated from a variety of tumor cells by different methods [21, 22]. At present, CD133, CD117, and Stro-1 are mostly used in osteosarcoma stem cell surface marker sorting. Among them, the combined sorting of CD117 and Stro-1 should be performed. Moreover, CD133 can be used as a cell marker to sort osteosarcoma cell lines MG-63, U-2OS and Saos-2 [23, 24]. In the experiment, the human CD133 antibody was used as the osteosarcoma stem cell surface markers by flow sorting. Three strains human MNNG/HOS, MG-63 and U-2 OS cells were sorted. The proportion of osteosarcoma stem cells fluctuate between 1 and 5%, with an average of 3%. The proportions of osteosarcoma stem cells were constant in different cell lines, which is comparable in the literature [23]. Any cell line can be used as the carrier for the next step experiment. Combined with the report in the literature, the osteosarcoma stem cell sorted from MNNG/HOS cells could enable the tumor form in non-obese diabetic (NOD) or severe combined immunodeficiency (SCID) and the tumor volume was larger [25]. Thus, the human osteosarcoma MNNG/HOS cell line was selected in the experiment.

Tumorsphere culture is the gold standard in the isolation and identification of stem cells. It is widely used in the identification of cancer stem cells after isoltion [23]. The CD133^+^ cell subsets were sorted out after CD133 antibody labeling. Then the tumorsphere culture experiment was performed, so as to further verify whether the CD133^+^ cell subsets were osteosarcoma stem cells. The tumorsphere culture found that the tumorsphere formed in the sorted CD133^+^ cell subsets with osteosarcoma stem cell characteristics after culture for 7 days. While no tumorsphere formed in the control group (CD133^−^ cells), this only confirms that the sorted CD133^+^ cells were the osteosarcoma stem cells. The tumor stem cells can form tumorspheres. The growth of CD133^+^ cell subsets was verified through growth curve assay (MTT), clone formation, osteogenic and adipogenic differentiation, nude mice tumor-forming capacity, and chemotherapy drug sensitivity test. Compared with CD133^−^ cell subsets in the control group, the OD values of CD133^+^ cell subsets were higher at different time-points in the logarithmic growth phase; after the same number of cells was added in the colony forming experiment, the colony-forming units were more and the colony area was larger; after osteogenic and adipogenic induction, the osteogenic and adipogenic differentiation was observed. The osteogenic and adipogenic genes *Bglap* and *adipoQ* were highly expressed. After 1.0 × 10^4^ cells were inoculated in nude mice, tumor formed. Twelve days later, 1.0 × 10^4^ CD133^−^ cells were then inoculated in the control group and extended to 4 weeks. No tumor growth was seen. After adding cisplatin, the survival rate was higher in equally distributed drug concentrations. The biological characteristics verified that the sorted CD133^+^ cell subsets were osteosarcoma stem cells. There were more colony-forming units. The potential of osteogenic and adipogenic differentiation was strong. The tumor-forming capacity in vivo was strong. The cells were resistant to the chemotherapy drugs. These were consistent with the characteristics of the sorted osteosarcoma stem cells in the literatures [23, 25]. CD133^−^ cells had no osteosarcoma stem cell-related characteristics, that was failure to form tumorspheres, weak ability of cell proliferation, less colony-forming units, no obvious differentiation potential or tumor-forming capacity in nude mice, and high sensitivity to chemotherapy drug cisplatin. In addition, the stem cell-related genes *Nanog* and *Oct4* were highly expressed in CD133^+^ cells by qPCR, which was about 2~4 times of CD133^-^ cells. The expressions of *TREX1* mRNA and protein in CD133^−^ cells of human osteosarcoma MNNG/HOS cells were significantly higher than those of CD133^+^ cells, suggesting that *TREX1* was lowly expressed in osteosarcoma stem cells and highly expressed in non-osteosarcoma stem cells, indicating that the expressions of *TREX1* gene and protein were closely related to osteosarcoma cell stem cell properties, also indicated that the expression of *TREX1* could indicate the stem cell characteristics of osteosarcoma cells, illustrating the proliferation and metastasis of osteosarcoma cells.

Patients in the metastatic group showed a low or no expression of TREX1, while the non-metastatic group expressed high TREX1 protein. Chemotherapy drug resistance is considered as the main reason for the poor prognosis in the metastatic group. Low expression of *TREX1* and resistance to cisplatin in CD133+ cells were observed indicating *TREX1* gene was related to the drug resistance of osteosarcoma, which was consistent with the previous report [26]. Thus, the expression of *TREX1* was closely related to the drug resistance, relapse and metastasis of osteosarcoma stem cells, and the expression of *TREX1* could indicate the prognosis of osteosarcoma.

## Conclusions

The expression of *TREX1* may be related to metastasis in patients with osteosarcoma. The expression of *TREX1* was closely related to the cytobiology characteristics of osteosarcoma stem cell. *TREX1* can play an important role in the occurrence and development processes. And *TREX1* is expected to become an effective new index for the evaluation of the prognosis. This was the preliminary experimental result based on the current clinical case, and a larger osteosarcoma sample validation was needed to confirm this result.

## Abbreviations

MTT: 3-(4,5-dimethylthiazol-2-yl)-2,5-diphenyltetrazolium bromide
NOD: Non-obese diabetic
PBS: Phosphate-buffered saline
SCID: Severe combined immunodeficiency
SR: Survival rate
*TREX1*: Three prime repair exonuclease 1
